# The study on copy number alteration of clear cell renal cancer in Chinese population

**DOI:** 10.7150/jca.33316

**Published:** 2020-01-01

**Authors:** Ning Zhang, Siteng Chen, Guangliang Jiang, Yishuo Wu, Jialiang Shao, Wennuan Liu, Xiang Wang, Rong Na, Jianfeng Xu

**Affiliations:** 1 Department of Urology, Ruijin Hospital, Shanghai Jiao Tong University School of Medicine, Shanghai, China; 2 Department of Urology, Shanghai Gerneral Hospital, Shanghai Jiao Tong University School of Medicine, Shanghai, China; 3 Department of Urology, Huashan Hospital, Fudan University, Shanghai, China; 4 Program for Personalized Cancer Care, Northshore University HealthSystem, Chicago, IL 60201, USA

**Keywords:** ccRCC, copy number alteration, Oncoscan, enrichment, gene burden, Chinese

## Abstract

**Objectives:** Copy number alteration (CNA) is one of the important genetic variations. Although there are many studies on renal cancer CNA, few studies are based on the Chinese population. In our study, our objective is to acquire the whole-genome CNA landscape in Chinese population and explore the tumor risk-associated functional genes in the CNA regions, by detecting whole-genome in the clear cell renal cancer (ccRCC) tissues.

**Methods:** We enrolled 35 formalin fixed paraffin embedded samples, which were processed by Oncoscan assay, and then acquired the data of whole-genome CNA. Then genes annotation and enrichment analyzing were processed. Furthermore, the gene burden and the affected bp (base pair) per Mbp (million bp) regions in whole-genome were analyzed by comparison of different T stage affected by CNA.

**Results:** We acquired the whole-genome CNA landscape by Oncoscan detection, and found out the high-frequency CNA regions which were not reported in previous studies, for example, 11P11, 22q11.23, 20q11.3 (PDRG1), and Xp22.33 so on. During the analyzing of genes annotation and enrichment, we found out some ccRCC functional genes in the CNA regions which might play a role in the biological process, for example, the copy number loss of DNA repair genes (TTC5、PARP2, etc.) and tumor suppressor genes (TADA3, VHL, BAP1, ERC2-IT1, etc.), the copy number gain of oncogenes (ABL2, MET, HUWE1, etc.) and Notch signal pathway genes (MDK, etc.). Besides, gene fusion (GSTTP and GSTTP2) was noticed at 22q11.23 which copy number loss occurred, and the frequency is 46%. And between the different T stage patients affected by CNA, the T2+T3 group carried more high-frequency CNA regions (*P*-value was 0.012).

**Conclusions:** In this study, the whole-genome ccRCC CNA landscape in Chinese population was acquired, a few functional genes and fusion genes were found out. However, a larger scale of samples is still needed to validate our results.

## Introduction

Renal cell cancer (RCC) represents 2%~3% of all cancers, and about 75% of RCC are clear cell renal cancer (ccRCC) 1. The estimated new cases and deaths were 63,990 and 14,400 in the US in 2017, and the number of new cases and deaths of renal cancer respectively were 15.6 per 100,000 and 3.9 per 100,000 men and women per year 2. In China, It was predicted that there would be about 66,800 newly diagnosed renal cancer cases and 23,400 deaths in 2015, namely 5.3 per 100,000 and 1.8 per 100,000 new cases and deaths 3. Despite the incidence rates of RCC stabilized during these years, due to the relatively high incidence, RCC has become one of the most important healthcare issues worldwide.

Copy number alteration (CNA) is one of the important genetic variations, whose regions of variation range from 1kbp to 1mbp, and is widely distributed in the human genome. Besides, some functional genes included in CNA maybe play a key role in the tumorigenesis, prognosis and response to drug treatment 4. For example, the copy number loss (CN loss) of tumor suppressor genes would affect the regulation of cell proliferation, leading to uncontrollable proliferation, and then cause cancer. In addition, the copy number gain (CN gain) of oncogenes also results in tumor occurrence, because of the accumulation of oncogenesis effect 5. Although there are many studies on RCC CNA, few studies are based on the Chinese population. In this study, our objective is to acquire the whole genome CNA landscape in Chinese population and explore the tumor risk-associated functional genes in the CNA regions, by detecting the whole genome in the ccRCC tissues.

## Materials and Methods

### Patients and tissues

All ccRCC specimens were from Huashan hospital, Fudan University. The inclusion criteria were: (1) Patients received radical nephrectomy or partial nephrectomy via open procedure or laparoscopic procedure because of kidney tumor; (2) the specimens were diagnosed with ccRCC by department of Pathology in our hospital (all the specimens were reviewed by the same group of pathologists.); (3) Clinical information was collected. Patients who were diagnosed with other types of kidney tumor, had missing clinical information were excluded. Finally, 35 FFPE (Formalin Fixed Paraffin Embedded) samples were included. The study was approved by the institutional review board of Huashan Hospital, Fudan University, Shanghai, China.

### The whole genome in the ccRCC and Statistical Analysis

The whole-genome DNA was isolated and purified from FFPE samples using QIAamp DNA FFPE Tissue Kit (QIAGEN), and was quantified using the Quant-iT™ PicoGreen® dsDNA Assay Kit (Life Technologies). Then, the DNA was processed by use of Oncoscan (Affymetrix Inc) assay, and array fluorescence intensity data (CEL files), generated by Affymetrix® GeneChip® Command Console® (AGCC) Software version 4.0 were processed using OncoScan® Console software to produce OSCHP files and a set of QC metrics (inclusion criteria: MAPD ≤0.3 and ndSNPQC ≥26). And then, the OSCHP files were analyzed and displayed by the software Nexus Express for Oncoscan 3.0 6. Finally, the visible data and whole genome CNA landscape were acquired, and the CNA frequency was calculated by Nexus Express for Oncoscan 3.0. Besides, genes annotation and enrichment analyzing were processed by the method of BP (Biological Process), GO (Gene ontology) in the DAVID database (the Database for Annotation, Visualization and Integrated Discovery), and the significant threshold was set as *P*-value <0.05. Then the functional genes related to biology process of oncogenesis in the CNA regions were further explored. Furthermore, the heatmap was conducted by R program (version 3.2.2). The SPSS (version 22) was used to analyze the gene burden and the affected base pair (bp) per million bp (Mbp) regions in the whole genome by comparison of different T stage affected by CNA.

## Results

### Baseline characteristics of the patients

In the Oncoscan assay, a total of 35 ccRCC samples were finally included in this study, of which 21 (60%) were male and 14 (40%) were female. The mean age of the cases was 57.6±9.6 and the mean body mass index (BMI) was 24.46±3.1. According to the TNM stage, there were 22 patients (63%) with T1a stage (tumor size ≤4cm), 6 patients (17%) with T1b stage (4cm< tumor size ≤7cm), 4 patients (11%) with T2a stage (7cm< tumor size ≤10cm), 2 patients (6%) with T2b stage (tumor size >10cm, but tumor confined to kidney), 1 patients (3%) with T3a stage (tumor grossly extends into the renal vein) (Table 1).

### Landscape of whole-genome CNA in Chinese population

**Figure 1:**
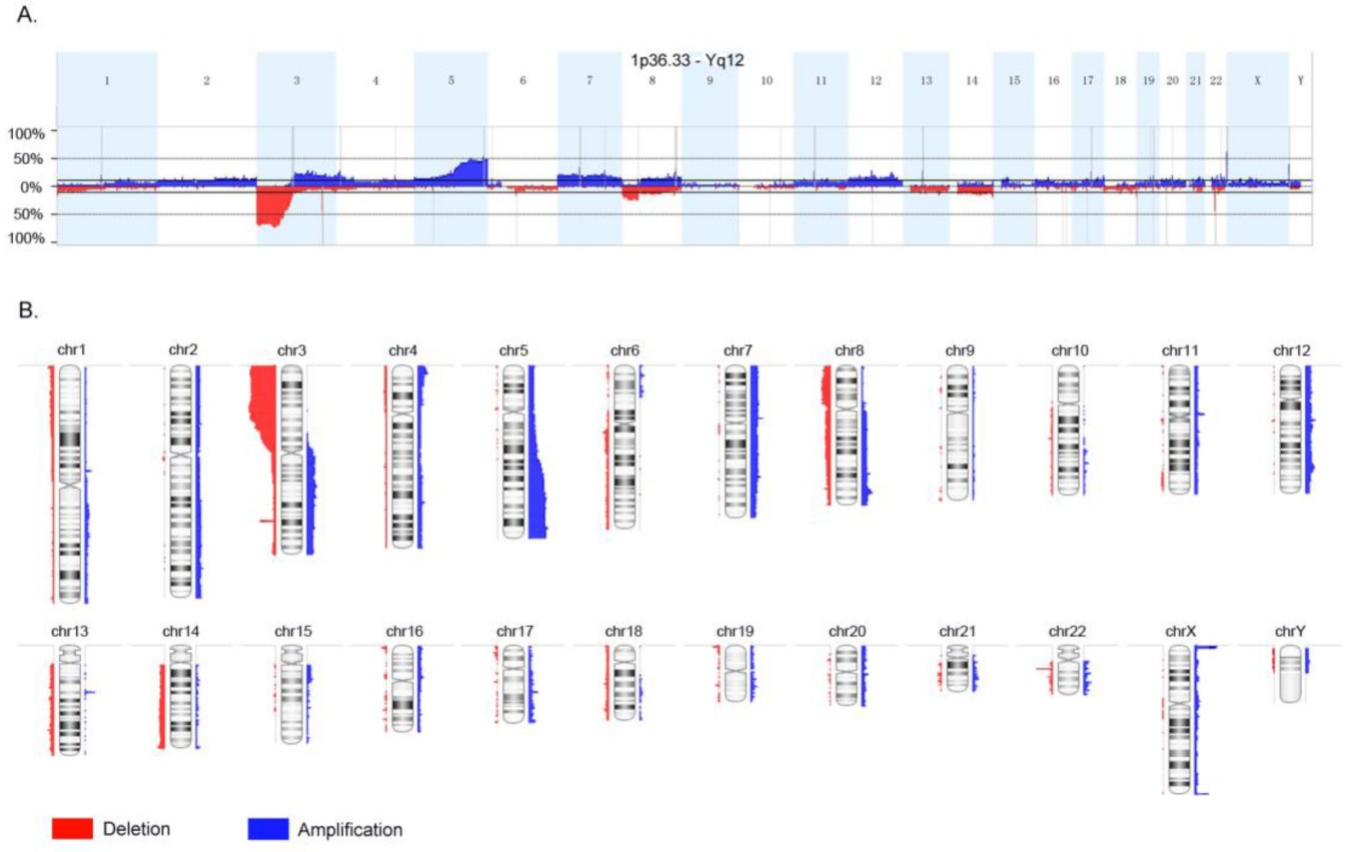
Landscape of whole-genome copy number alteration (CNA). The red and blue areas respectively represented copy number loss and copy number gain, and the statue of whole-genome CNA in 24 chromosomes of 35 clear cell renal cancer samples was observed clearly.

**Figure 2:**
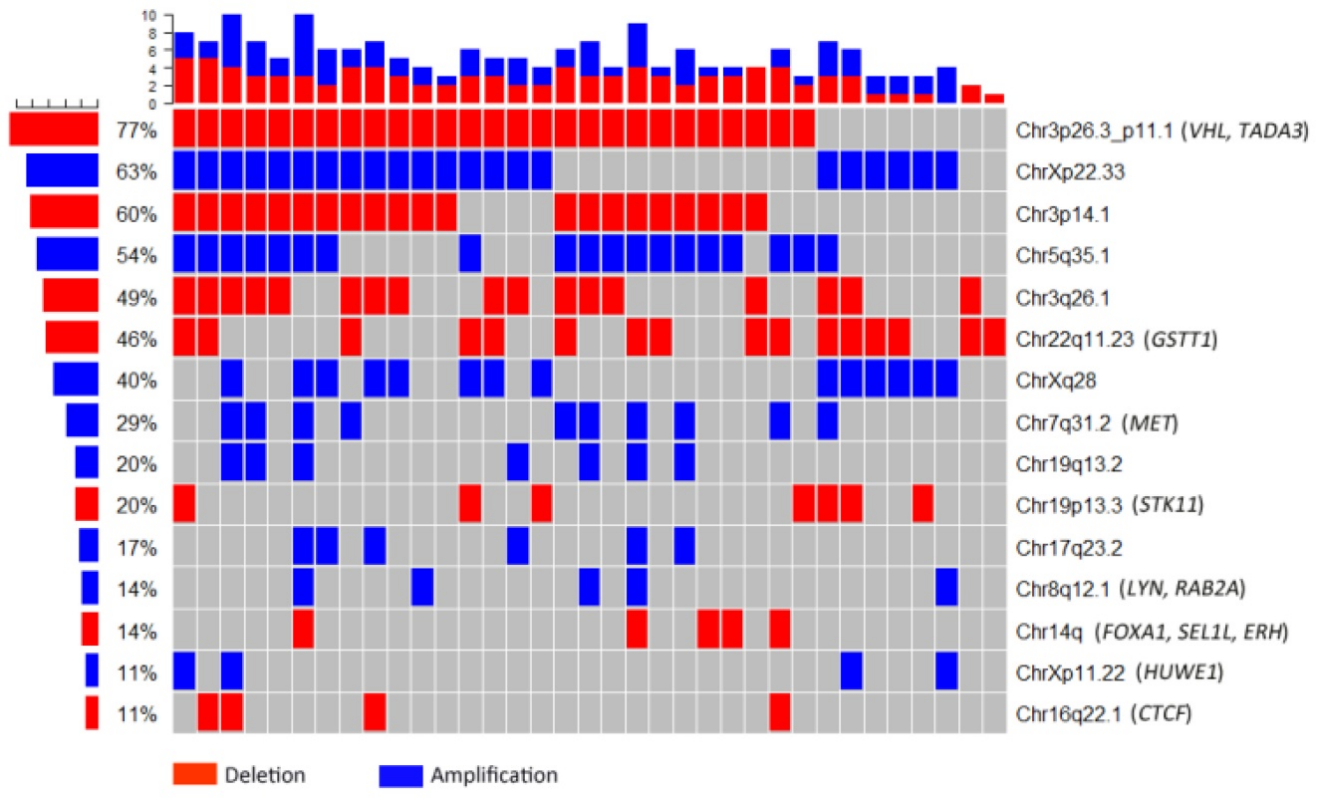
Heatmap based on copy number alteration regions of high frequency and representative functional genes. The red and blue areas respectively represented copy number loss and copy number gain. The left histograms showed percentages of affected samples.

The whole-genome CNA were detected in 35 ccRCC FFPE samples using Oncoscan assay. The software Nexus Express for Oncoscan 3.0 was used to analyze the raw data, generate the visible data, and depict the landscape of whole-genome CNA (Fig. 1A and Fig. 1B). In this CNA landscape, the red and blue areas respectively represented CN loss and CN gain, and the statue of whole-genome CNA in 24 chromosomes of 35 ccRCC samples was observed clearly. For example, we could notice the obvious CN loss in the whole chromosome 3p, partial chromosome 8p and chromosome 22q, and the frequencies of occurrence were high. Besides, we could notice the CN gain regions of high frequencies in chromosome 5q, chromosome Xq and Xp. Furthermore, the heatmap (Oncoprint) was depicted based on CNA regions of high frequency and representative functional genes (Fig. 2). This heatmap showed CN losses (blue) and gains (red) in 35 samples with Ocoscan assay data, and the affected genes in each locus that harbor CNA were listed in the parentheses. In this heatmap, the left histograms showed percentages of affected samples.

Based on the outcomes of Ocoscan assay in our study, we chose some CNA regions which had high frequency (≥20%) to make comparison with the Cancer Genome Atlas (TCGA) database on CNA associated with ccRCC (Table 2). In the first four columns of Table 2, we listed the high-frequency CNA regions we found from Ocoscan assay, among which the CN gain in 11p11 (MDK), 22q11.23 (PLA2G6), Xp22.33 (XGY3), and the CN loss in 20q11.3 (PDRG1), 16p11.2-p11.1 (SMG1P2), were found for the first time. Compared with TCGA database, the CNA frequency of some regions in Chinese was obviously higher, for example, 3p25.3 (VHL), 5q35.3 (SQSTM1), 13q21 (RB1), 17q21.33 (ABCC3), and Xq28 (SPRY3) so on. So the functional genes located in these CNA regions had a potential to become the RCC biomarkers. In the Supplementary Table 1, more CNA regions in Chinse were compared with TCGA cohort.

### Genes functional annotation and enrichment

**Figure 3:**
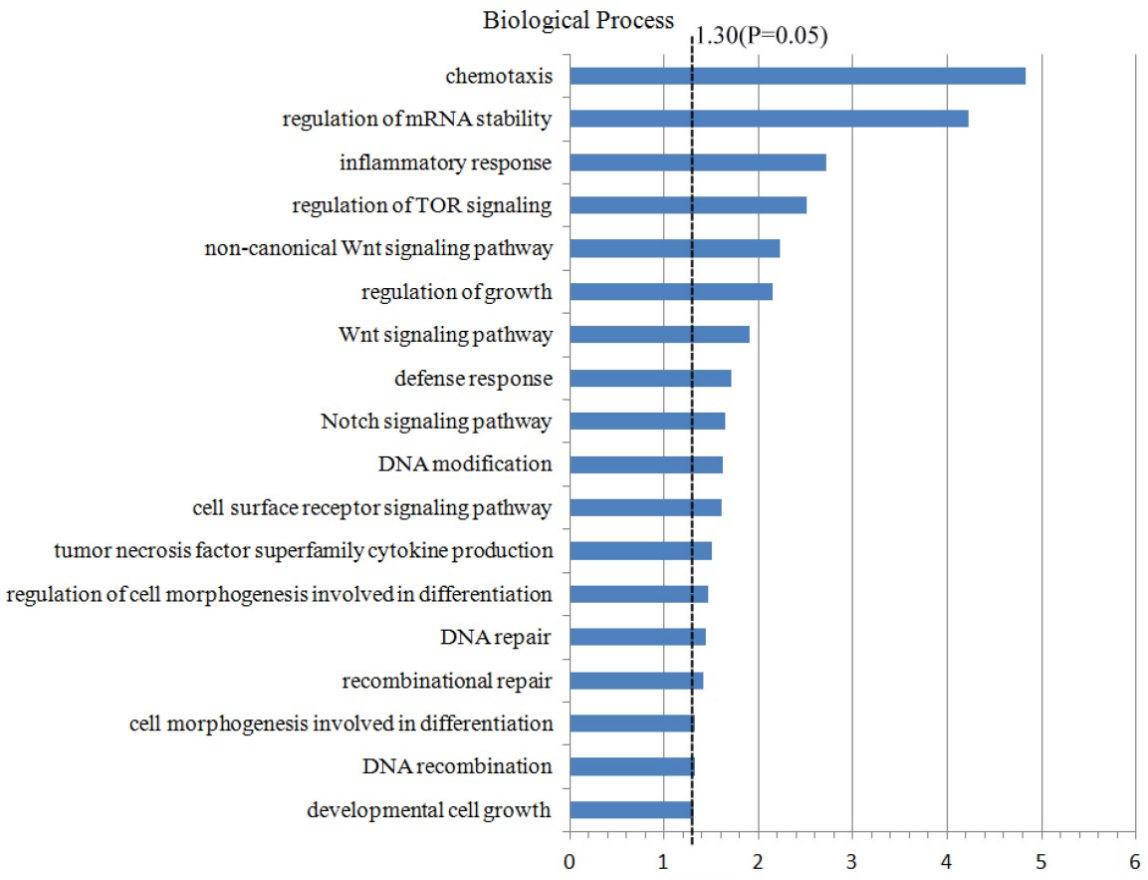
Genes functional annotation and enrichment. 4437 genes which were located in the CNA regions with the frequency ≥10% were included in the Database for Annotation, Visualization and Integrated Discovery to analyze genes annotation and enrichment.

For the further study on the CNA regions and the genes located in these regions, 4437 genes which were located in the CNA regions (the frequency ≥10%) were included to conduct the analyzing of genes annotation and enrichment by the method of biological process in the Database for Annotation, Visualization and Integrated Discovery (DAVID). During this process, we set the significant threshold as *P*-value <0.05, and acquired a few potential biological processes and functional genes related to the oncogenesis and tumor progression (Fig. 3). For example, the functional genes which were enriched in the Wnt signal pathway (PSME2, PSMB11, PSMB5) and Notch signal pathway (FOXA1, MMP14, MDK), DNA repair pathway (REC8, DDB2, FAN1, TTC5, etc.), DNA modification pathway (DNMT3A, ASCC1, CTCF, etc.). Furthermore, these functional genes in different pathways which were significantly enriched were compared with TCGA database (Table 3). Finally, some higher-frequency functional genes and new-found genes were screened out, which might play a role in the oncogenesis and tumor progression, and be the tumor-related risk factors.

### Oncogenes, Tumor suppressor genes and Fusion genes

Through much deeper analyzing 4437 genes which were located in the CNA regions (the frequency ≥10%), we explored and selected out 23 tumor suppressor genes located in CN loss regions (TADA3, VHL, PLCD1, CSRNP1, LZTFL1, LRRC2, NAT6, TUSC2, RASSF1, PCBP4, BAP1, ERC2-IT1, PTPRG, FOXP1, etc.), and 10 oncogenes (ABL2, RAB10, ADGRA3, BRAF, MET, LYN, MOS, RAB2A, RNF139, HUWE1) which were located in CN gain regions (Table 4). There were still no studies on these functional genes associated with ccRCC, except for three genes (VHL, BAP1, MET). Besides, among these functional genes, the CNA of tumor suppressor gene (ERC2-IT1) and oncogenes (RAB10, ADGRA3) were not reported either in TCGA database. Interestingly, except for some higher-frequency genes and no-reporter genes, the opposite results of some CNA genes between Oncoscan assay and TCGA were also noticed, such as TSC2, RNF40 and HUWE1.

**Figure 4:**
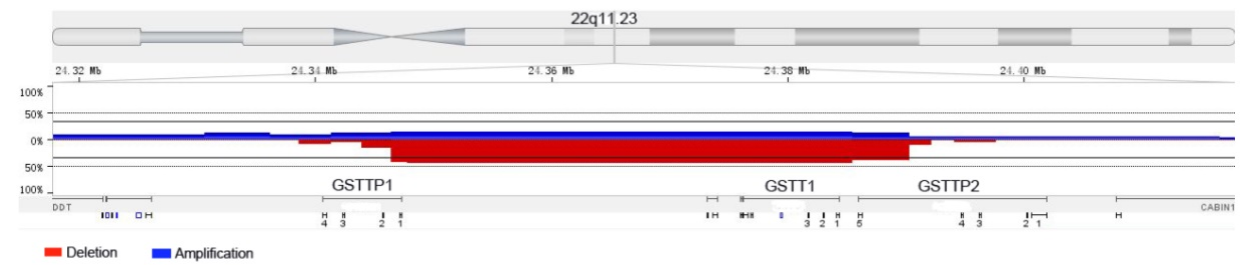
The relatively high-frequency gene fusion in 22q11.23. The red and blue areas respectively represented copy number loss and copy number gain. Two genes fusion (GSTTP1 and GSTTP2) located at two sides of copy number loss fragment were observed.

To explore the fusion genes, we chose a few high-frequency CN loss regions for further analyzing, such as 3p, 3q25.33-q26.1 and 22q11.23. Although we did not find the phenomenon of gene fusion in 3p and 3q25.33-q26.1, we observed the relatively high-frequency gene fusion in 22q11.23 (Fig. 4). When the CN loss of certain fragment in 22q11.23 occurred, two genes fusion (GSTTP1 and GSTTP2) which were located at two sides of CN loss fragment happened. And in 35 samples, gene fusion occurred in 16 samples (46%).

### The comparison between different T stages affected by CNA

In the Table 5, the statistical difference among different T stages was calculated on some high-frequency CNA regions. Although there were no significance in the group of CN loss, CN gain, LOH (loss of heterozygosity), the significant difference of total CNA between T1 group and T2+T3 group was noticed (*P*-value =0.012), which mean T2+T3 group carried more high-frequency CNA regions (Supplementary Table 2). In the Supplementary Table 3, we calculated the number of genes affected by CNA in every T stage patient, namely gene burden, and compared the difference between T1 and T2+T3 stage. But there were no significance in the group of CN loss, CN gain, LOH, and total CNA (*P*-value > 0.05). As the Supplementary Table 4 showed, the affected bp per Mbp regions in the whole genome (about 3000Mbp) of every patient were analyzed, and the significance on different T stages in the group of CN loss, CN gain, LOH, and total CNA were not found out (*P*-value > 0.05).

## Discussion

As far as we know, it is the first time that the whole-genome CNA in ccRCC was reported in the Chinese population, and the landscape of whole-genome CNA in ccRCC was acquired. In this study, we found out a few CNA regions which were not reported in previous studies, and discovered some new functional genes associated with ccRCC which had definite biological meaning. For example, the CN loss of tumor suppressor genes and DNA repair genes, the CN gain of oncogenes, fusion genes, etc. Commonly, gene dose effect, loss of gene function, and gene fusion were three likely mechanisms in CNA that would lead to the disease 7. Therefore, these functional genes had potential to become the biomarkers of ccRCC, and were used for early diagnosis and guidance of follow-up.

In previous studies, researchers acquired the somatic CNAs by detecting 385 ccRCC samples with gene sequencing 8. They discovered a few high-frequency CNA regions and functional genes, for example, the CN loss in 3p21.1, 14q24, 9p21.3 (CDKN2A), 6q26 (QKI), 8p11, 10q23 (PTEN), 1p26, 4q35, 13q21 (RB1), 15q21, 2q37, and the CN gain in 5q35, 8q24 (MYC), 3q26 (MECOM), 1q32 (MDM4). It was supposed that CN loss of tumor suppressor genes (CDKN2A, PTEN, RB1) and CN gain of oncogene (MYC) could be closely associated with tumorigenesis 8,9. In another study, it reported some high-frequency CNA regions and functional genes by whole-genome sequencing in 85 ccRCC samples, for example, EPM2AIP1 and SUSD5 at 3p22-p24, APEH, SETD2 and SMARCC at 13p21.31, SLC6A6 and VHL at 3p25, YLPM1 at 14q24.3, C14orf39 at 14q23, PNLIP at 10q25, DOCK2 at 5q35.1, KAT5 at 11q13.1, CHRNB2 and ECM1 at 1q21.2, AKAP9 and MUC17 at 7q11-q22 10. It was reported that ccRCC was characterized by loss of function of the tumor suppressor VHL and activation of hypoxia-inducible transcription factors (HIFs), and the VHL gene product (pVHL) targeted HIFs transcription factors for proteasomal degradation, and then increased HIF2a promotes pVHL-defective tumorigenesis 11-13. There was a study that showed 5q amplification led to overexpression of the SQSTM1 oncogene in ccRCC lines and tumors, and the gene product of which, p62, regulated known renal cancer suppressor genes such as VHL, TSC1and TSC2 14,15. Therefore, SQSTM1 was regarded as a pathogenic target of 5q CN gains in renal cancer. Besides, there were studies suggested that the CN loss of tumor suppressor CDKNA2 at 9p and the CN gain of oncogene MET at 7q31.1 were closely related to RCC, having the potential predictive value to guide targeted therapy 16,17.

In the Table 3, we listed the new-found functional genes that were not reported before in ccRCC, including DNA repair genes, DNA modification genes, Notch signal pathway genes, and Wnt signal pathway genes. The previous studies validated that the alterations in DNA repair genes were closely associated with tumorigenesis and prognosis, including renal cancer 18,19. In DNA modification genes we found, several studies showed that CN loss of CTCF at 16q22.1 and its low expression occurred in breast cancer and Wilms tumor of the kidney 20,21. A study demonstrated that CNA led to Notch pathway activation in ccRCC, and relevant gene products were also overexpressed and associated with gene amplification in distinct ccRCC samples 22. The high-frequency CN gain of MDK at 11p11.2 in our study might validate these outcomes. Moreover, another study indicated that lower FOXA1 expression might be a marker of aggressive disease in upper tract urothelial carcinoma undergoing radical nephroureterectomy 23. This outcome might be an evidence to prove that CN loss of FOXA1 at 14q11.2 was associated with ccRCC in our study. There was a research indicated that the low expression of the Wnt signal pathway gene (Wnt7A) was detected in the majority of ccRCC, and might have tumor suppressor properties 24. Therefore, this result might be also a proof to verify that CN loss of PSME2, PSMB11, PSMB5 was associated with ccRCC in our study.

In the Table 4, we made a list of functional genes, including 23 tumor suppressor genes located in CN loss regions and 10 oncogenes at CN gain regions. The previous studies had validated that CN loss of tumor suppressors VHL, BAP1, and CN gain of oncogene MET might play a role in tumorigenesis and progression 16,25. For the rest of genes listed in Table 4, there were still no studies on ccRCC CNA in these functional genes. In short, all they had the potential to become markers of ccRCC. For the fusion genes, although both GSTTP1 and GSTTP2 were pseudogene, GSTTP1 still took part in Glutathione metabolism. As we know, the metabolic change was one of the important characters of tumor cells. Moreover, more and more researches revealed that pseudogene could regulate normal genes or express a part of protein, and the alteration in pseudogene might result in cancer 26. Actually, we should further design the primers corresponding to GSTTP1 and GSTTP2, to validate the existing of gene fusion by PCR test.

In addition, the CNA frequency of some regions in Chinese was obviously higher when compared with TCGA database. Some genes which were found to be deleted in the TCGA database are amplified in our Ocoscan assay (e.g., EGFR, etc.), while some genes which were found to be amplified in the TCGA are deleted in our study (e.g., TSC2, etc.). However, it is hard to conclude whether the discrepancy is indeed due to race (Chinese population versus European Americans) or merely due to small study size. A future larger cohort should also be conducted to validate these findings.

Several limitations of the current study should be noted. (1) The sample size is relatively small in this study. It is hard to conclude whether the discrepancy is indeed due to race (Chinese population versus European Americans) or merely due to small size. It would be more persuasive to conduct Oncoscan assay on more samples. (2) The differences which had CNA regions or genes between different T stages were not detected, probably because of the small sample size which limited the statistic significance. (3) For the functional genes we found in this study, the validation by low-throughput method (MLPA, HRM-PCR, etc.) should be done in larger scale of samples. (4) The study on expression of functional genes is lacking, we will conduct the related experiment to validate the function of these genes in the next step.

## Conclusion

In this study, we acquired the whole-genome ccRCC CNA landscape in the Chinese population, discovered a few functional genes and fusion genes related to ccRCC in the CNA regions which maybe play a role in the biological process of tumor tumorigenesis. However, the larger scale of samples still is needed to validate our results.

## Supplementary Material

Supplementary figures and tables.

## Figures and Tables

**Table 1:** Demographic and clinical information of study population

Variables		ccRCC (n=35)
No. of cases		35
Age (yr), Median (range)		55 (40-85)
**Gender (n, %)**		
	Male	21 (60%)
	Female	14 (40%)
Tumor size (cm), Median (Range)		3.5 (2-13)
**T Stage (n, %)**		
	T1a	22 (63%)
	T1b	6 (17%)
	T2a	4 (11%)
	T2b	2 (6%)
	T3a	1 (3%)
**N stage (n, %)**		
	N0	35 (100%)
	N1	0
**M stage (n, %)**		
	M0	35 (100%)
	M1	0
**Furhman (n, %)**		
	I	1 (3%)
	II	17 (48%)
	III	3 (9%)
	IV	0
	Missing	14 (40%)

ccRCC, clear cell renal cancer; yr, year.

**Table 2:** CNAs of ccRCC with highest frequencies (≥20%) in Chinese and the comparison with TCGA cohort (mainly European Americans)

Chr	Region	Chinese (n=35)			TCGA (n=537)			Affected Genes
		Event	Frequency		Event	Frequency		
3	p26.2	Del	48.57%		Del	91%		MECOM
	p25.3	Del	71.43%		Del	11%		VHL
	p22-p21	Del	74.29%		Del	89-92%		BAP1, SETD2
	p12.1	Del	31.43%		Del	51%		CADM2
	q21	Amp	31.43%		Amp	0.4%		CNBP
5	q31.2	Amp	51.43%		Amp	14%		EGR1, PKD2L2
	q35.3	Amp	48.57%		Amp	15%		SQSTM1
7	q22.1	Amp	20.00%		Amp	0.7%		RADIL, TRRAP, ZAN
8	q24.21	Amp	25.71%		Amp	15%		MYC
11	p11	Amp	31.43%		-	-		OR4C12,MDK
13	q21	Del	22.86%		Del	0.20%		RB1
14	q	Del	51.43%		Del	45%		HIF1A,NRXN3
16	p11.2-p11.1	LOH	100%		-	-		SMG1P2
17	q21.33	Amp	31.43%		Amp	0.40%		ABCC3
19	q13.2	Amp	28.57%		Amp	0.20%		WTIP
20	q11.3	Del	20.00%		-	-		PDRG1
22	q11.23	Amp	45.71%		-	-		PLA2G6
X	p22.33	Amp	62.86%		-	-		XGY2
	q28	Amp	40%		Amp	0.80%		SPRY3

CNA, copy number alteration; ccRCC, clear cell renal cancer; TCGA, the Cancer Genome Atlas; Chr, chromosome; Del, delete; LOH, loss of heterozygosity; Amp, amplification.

**Table 3:** CN status of the genes in different pathways that are significantly enriched

Pathways	Related genes	Chinese			TCGA	
		Event	Frequency		Event	Frequency
Regulation of mRNA stability	EXOSC7	Del	71.43%		Del	10%
	PRKCD	Del	71.43%		Del	9%
	TRIM71	Del	68.57%		Del	10%
	HNRNPA0	Amp	45.71%		Amp	13%
	METTL3	Del	11.42%		-	-
	PSME1	Del	11.42%		-	-
DNA repair	DDB2	Amp	31.43%		-	-
	PARP2	Del	11.42%		-	-
	TTC5	Del	11.42%		-	-
	APEX1	Del	11.42%		-	-
	SUPT16H	Del	11.42%		-	-
	REC8	Del	11.42%		-	-
	FAN1	Amp	31.43%		-	-
	EGFR	Amp	37.14%		Del	0.20%
	KIF22	LOH	34.29%		Amp	0.20%
	PPP4C	LOH	48.57%		Amp	0.20%
	INO80E	LOH	42.86%		Amp	0.20%
	PAGR1	LOH	34.29%		Amp	0.20%
DNA modification	DNMT3A	Amp	11.42%		-	-
	EHMT2	Amp	11.42%		-	-
	ASCC1	Del	11.42%		-	-
	CCNB1IP1	Del	11.42%		-	-
	CTCF	Del	11.42%		-	-
Notch signaling pathway	MDK	Amp	31.43%		-	-
	MMP14	Del	11.42%		-	-
	FOXA1	Del	11.42%		-	-
TOR signaling pathway	TSC2	Del	14.29%		Amp	0.20%
	TELO2	LOH	11.43%		Amp	0.20%
	WDR24	LOH	11.43%		Amp	0.20%
	MLST8	Del	14.29%		Amp	0.20%
Wnt signaling pathway	PSMB5	Del	11.42%		-	-
	PSMB11	Del	11.42%		-	-
	PSME2	Del	11.42%		-	-
Tumor necrosis factor superfamily	PYCARD	LOH	65.71%		Amp	0.20%
	GHRL	Del	68.57%		Del	11%
	CCR2	Del	74.29%		Del	10%
	MYD88	Del	71.43%		Del	10%
	LTF	Del	74.29%		Del	11%
Inflammatory response	TUSC2	Del	68.57%		Del	10%
	PPARG	Del	68.57%		Del	11%
	IL17RC	Del	71.43%		Del	11%
	CCRL2	Del	74.29%		Del	11%
	HIF1A	Del	14.29%		Del	0.60%
	LTB4R	Del	11.42%		-	-
	IL25	Del	11.42%		-	-
	LTB4R2	Del	11.42%		-	-
	NFATC4	Del	11.42%		-	-

CN, copy number; TCGA, the Cancer Genome Atlas; Del, delete; LOH, loss of heterozygosity; Amp, amplification.

**Table 4:** Tumor suppressors and Oncogenes associated with ccRCC in this study

Function	Genes	Region	Chinese		TCGA	
			Event	Frequency	Event	Frequency
Tumor suppressors(23)	TADA3	3p25.3	Del	71.43%	Del	11.00%
	VHL*	3p25.3	Del	71.43%	Del	11.00%
	PLCD1	3p22.2	Del	71.43%	Del	11.00%
	CSRNP1	3p22.2	Del	71.43%	Del	11.00%
	LZTFL1	3p21.31	Del	74.29%	Del	11.00%
	LRRC2	3p21.31	Del	74.29%	Del	11.00%
	SEMA3B	3p21.31	Del	66.57%	Del	11.00%
	NAT6	3p21.31	Del	68.57%	Del	11.00%
	TUSC2	3p21.31	Del	68.57%	Del	11.00%
	RASSF1	3p21.31	Del	68.57%	Del	11.00%
	PCBP4	3p21.2	Del	71.43%	Del	11.00%
	BAP1*	3p21.1	Del	71.43%	Del	10.00%
	ERC2-IT1	3p14.3	Del	71.43%	-	-
	PTPRG	3p14.2	Del	57.14%	Del	5.00%
	FOXP1	3p13	Del	42.86%	Del	2.70%
	RBM5	3p21.31	Del	68.57%	Del	10%
	LATS2	13q12.11	Del	17.14%	Del	0.20%
	RNF6	13q12.13	Del	17.14%	Del	0.20%
	DLK1	14q32.2	Del	11.43%	Del	0.40%
	MEG3	14q32.2	Del	11.43%	Del	0.40%
	TSC2	16p13.3	Del	14.29%	Amp	0.20%
	RNF40	16p11.2	LOH	57.14%	Amp	0.20%
	STK11	19p13.3	Del	20.00%	Del	0.20%
Oncogenes (10)	ABL2	1q25.2	Amp	14.29%	Amp	0.80%
	RAB10	2p23.3	Amp	11.43%	-	-
	ADGRA3	4p15.2	Amp	11.43%	-	-
	BRAF	7q34	Amp	17.14%	Amp	0.90%
	MET*	7q31.2	Amp	28.57%	Amp	0.90%
	LYN	8q12.1	Amp	14.29%	Amp	0.40%
	MOS	8q12.1	Amp	14.29%	Amp	0.40%
	RAB2A	8q12.1	Amp	14.29%	Amp	0.40%
	RNF139	8q24.13	Amp	14.29%	Amp	0.80%
	HUWE1	Xp11.22	Amp	11.43%	Del	0.60%

*: The genes associated with renal cancer have been reported in previous studies; -: not available; ccRCC, clear cell renal cancer; TCGA, the Cancer Genome Atlas; Del, delete; LOH, loss of heterozygosity; Amp, amplification.

**Table 5:** CNA burden in ccRCC patients.

Event	Average level (%)	No. of cases with high level of CAN (n, %)		
		T1 (n=28)	T2+T3 (N=7)	P-value
Amplification	2.91%	13 (46.43%)	4 (57.14%)	0.69
Deletion	2.49%	13 (46.43%)	4 (57.14%)	0.69
Amplification and deletion	5.40%	13 (46.43%)	7 (100%)	0.012*
Loss of heterozygosity	5.08%	13 (46.43%)	4 (57.14%)	0.69

CNA, copy number alteration; ccRCC, clear cell renal cancer; *: P-value < 0.05.

## Abbreviations

RCC: renal cell cancer
ccRCC: clear cell renal cancer
CNA: copy number alteration
bp: base pair
Mbp: million bp
CN loss: copy number loss
CN gain: copy number gain
BP: biological process
GO: gene ontology
BMI: body mass index
TCGA: the Cancer Genome Atlas
DAVID: The Database for Annotation, Visualization and Integrated Discovery
Chr: chromosome
Del: delete
LOH: loss of heterozygosity
Amp: amplification

## References

[1] Ljungberg B, Bensalah K, Canfield S, Dabestani S, Hofmann F, Hora M (2015). EAU guidelines on renal cell carcinoma: 2014 update. European urology.

[2] Siegel RL, Miller KD, Jemal A (2017). Cancer Statistics, 2017. CA: a cancer journal for clinicians.

[3] Chen W, Zheng R, Baade PD, Zhang S, Zeng H, Bray F (2016). Cancer statistics in China, 2015. CA: a cancer journal for clinicians.

[4] Arai E, Kanai Y (2010). Genetic and epigenetic alterations during renal carcinogenesis. International journal of clinical and experimental pathology.

[5] Stranger BE, Forrest MS, Dunning M, Ingle CE, Beazley C, Thorne N (2007). Relative impact of nucleotide and copy number variation on gene expression phenotypes. Science.

[6] Foster JM, Oumie A, Togneri FS, Vasques FR, Hau D, Taylor M (2015). Cross-laboratory validation of the OncoScan(R) FFPE Assay, a multiplex tool for whole genome tumour profiling. BMC medical genomics.

[7] Kuiper RP, Ligtenberg MJ, Hoogerbrugge N, Geurts van Kessel A (2010). Germline copy number variation and cancer risk. Current opinion in genetics & development.

[8] Network CGAR (2013). Comprehensive molecular characterization of clear cell renal cell carcinoma. Nature.

[9] O'Leary NA, Wright MW, Brister JR, Ciufo S, Haddad D, McVeigh R (2016). Reference sequence (RefSeq) database at NCBI: current status, taxonomic expansion, and functional annotation. Nucleic acids research.

[10] Scelo G, Riazalhosseini Y, Greger L, Letourneau L, Gonzalez-Porta M, Wozniak MB (2014). Variation in genomic landscape of clear cell renal cell carcinoma across Europe. Nature communications.

[11] Damjanovic SS, Ilic BB, Beleslin Cokic BB, Antic JA, Bankovic JZ, Milicevic IT (2016). Tuberous sclerosis complex protein 1 expression is affected by VHL Gene alterations and HIF-1alpha production in sporadic clear-cell renal cell carcinoma. Experimental and molecular pathology.

[12] Grampp S, Platt JL, Lauer V, Salama R, Kranz F, Neumann VK (2016). Genetic variation at the 8q24.21 renal cancer susceptibility locus affects HIF binding to a MYC enhancer. Nature communications.

[13] Shen C, Kaelin WG Jr (2013). The VHL/HIF axis in clear cell renal carcinoma. Seminars in cancer biology.

[14] Li L, Shen C, Nakamura E, Ando K, Signoretti S, Beroukhim R (2013). SQSTM1 is a pathogenic target of 5q copy number gains in kidney cancer. Cancer cell.

[15] Kozlowski P, Roberts P, Dabora S, Franz D, Bissler J, Northrup H (2007). Identification of 54 large deletions/duplications in TSC1 and TSC2 using MLPA, and genotype-phenotype correlations. Human genetics.

[16] Macher-Goeppinger S, Keith M, Endris V, Penzel R, Tagscherer KE, Pahernik S (2017). MET expression and copy number status in clear-cell renal cell carcinoma: prognostic value and potential predictive marker. Oncotarget.

[17] El-Mokadem I, Kidd T, Pratt N, Fleming S, Nabi G (2016). Tumour suppressor gene (CDKNA2) status on chromosome 9p in resected renal tissue improves prognosis of localised kidney cancer. Oncotarget.

[18] Margulis V, Lin J, Yang H, Wang W, Wood CG, Wu X (2008). Genetic susceptibility to renal cell carcinoma: the role of DNA double-strand break repair pathway. Cancer epidemiology, biomarkers & prevention.

[19] Akhmadishina LZ, Giliazova IR, Kutlyeva LR, Korytina GF, Kochetova OV, Urmantsev MF (2014). [DNA repair XRCC1, XPD genes polymorphism as associated with the development of bladder cancer and renal cell carcinoma]. Genetika.

[20] Mummert SK, Lobanenkov VA, Feinberg AP (2005). Association of chromosome arm 16q loss with loss of imprinting of insulin-like growth factor-II in Wilms tumor. Genes, chromosomes & cancer.

[21] Green AR, Krivinskas S, Young P, Rakha EA, Paish EC, Powe DG (2009). Loss of expression of chromosome 16q genes DPEP1 and CTCF in lobular carcinoma in situ of the breast. Breast cancer research and treatment.

[22] Bhagat TD, Zou Y, Huang S, Park J, Palmer MB, Hu C (2017). Notch Pathway Is Activated via Genetic and Epigenetic Alterations and Is a Therapeutic Target in Clear Cell Renal Cancer. The Journal of biological chemistry.

[23] Raman JD, Warrick JI, Caruso C, Yang Z, Shuman L, Bruggeman RD (2016). Altered Expression of the Transcription Factor Forkhead Box A1 (FOXA1) Is Associated With Poor Prognosis in Urothelial Carcinoma of the Upper Urinary Tract. Urology.

[24] Kondratov AG, Kvasha SM, Stoliar LA, Romanenko AM, Zgonnyk YM, Gordiyuk VV (2012). Alterations of the WNT7A gene in clear cell renal cell carcinomas. PloS one.

[25] Gao W, Li W, Xiao T, Liu XS, Kaelin WG Jr (2017). Inactivation of the PBRM1 tumor suppressor gene amplifies the HIF-response in VHL-/- clear cell renal carcinoma. Proceedings of the National Academy of Sciences of the United States of America.

[26] Hirotsune S, Yoshida N, Chen A, Garrett L, Sugiyama F, Takahashi S (2003). An expressed pseudogene regulates the messenger-RNA stability of its homologous coding gene. Nature.

