# Supplementary material for: The study on copy number alteration of clear cell renal cancer in Chinese population
Source: J Cancer. 2020 Jan 1;11(1):16–24. doi: 10.7150/jca.33316 (PMC6930398; doi:10.7150/jca.33316)
Supplement: Supplementary file 1 — Supplementary figures and tables. [file jcav11p0016s1.pdf]

Supplementary Table 1 CNAs of ccRCC in Chinese cohort and the comparison with TCGA cohort (mainly European Americans)

Chr	Region	Chinese （n=35）		TCGA (n=537)		Affected Genes
		Event	Frequency	Event	Frequency	
1	p36.22	Del	11.43%	Del	16.00%	
1	p36.13	Del	11.43%	Del	16.00%	
1	p35.3	LOH	42.86%	Del	14.00%	
1	p31.1	LOH	31.43%	Del	12.00%	
1	q21.3	Amp	11.43%	Amp	14.00%	
1	q32.1	Amp	8.57%	Amp	14.00%	*MDM4*
1	q41	Amp	11.43%	Del	5.00%	
1	q42.3	Del	2.86%	Del	5.00%	
2	q37.1	Amp	14.29%	Del	8.00%	
2	q33.1	Amp	14.29%	Amp	16.00%	
3	p26.3	Del	68.57%	Amp	2.00%	
3	p26.1	Del	71.43%	Del	91.00%	
3	p21.1	Del	71.43%	Del	92.00%	
3	p14.2	Del	62.86%	Del	80.00%	
3	p14.1	Del	60.00%	Del	71.00%	
3	p12.3	Del	45.71%	Del	60.00%	
3	p12.1	Del	31.43%	Del	51.00%	*CADM2*
3	p11.2	Del	20.00%	Del	47.00%	
3	q11.2	Amp	25.71%	Del	32.00%	
3	q12.1	Amp	22.86%	Del	31.00%	
3	q26.2	Amp	25.71%	Amp	15.00%	*PRKCI, MECOM*
4	q24	Amp	11.43%	Del	16.00%	
4	q32.1	Amp	11.43%	Amp	3.00%	
4	q35.2	Amp	14.29%	Del	16.00%	
5	q15	Amp	28.57%	Amp	44.00%	
5	q21.1	Amp	37.14%	Amp	50.00%	
5	q35.2	Amp	48.57%	Amp	67.00%	
6	q15	Del	11.43%	Del	24.00%	
6	q26	Del	5.71%	Del	28.00%	*QKI*
6	q27	Del	8.57%	Del	28.00%	
7	q11.22	Amp	20.00%	Amp	34.00%	
7	q31.1	Amp	28.57%	Del	1.00%	
7	q36.2	Amp	17.14%	Amp	34.00%	
8	p23.2	Del	22.86%	Del	31.00%	
8	p21.1	Del	22.86%	Del	30.00%	
8	p11.21	Del	20.00%	Del	25.00%	
8	q24.21	Amp	25.71%	Amp	15.00%	*MYC*
9	p24.1	Amp	11.43%	Del	3%-31%	*PTPRD,JAK2*
9	p21.3	Del	5.71%	Del	32.00%	*CDKN2A*
10	p15.3	Amp	2.86%	Del	12.00%	

10	q23.31	Amp	11.43%	Del	18.00%	
10	q26.2	Del	2.86%	Del	18.00%	
11	q23.3	Amp	11.43%	Del	6.00%	
13	q12.13	Del	8.57%	Del	13.00%	
13	q33.3	Del	14.29%	Del	14.00%	
14	q13.1	Del	11.43%	Amp	3.00%	
14	q24.3	Del	20.00%	Del	45.00%	*NRXN3*
15	q21.1	Amp	11.43%	Del	8.00%	
15	q26.1	Amp	0.00%	Amp	5.00%	*IDH2*
17	q12	Del/Amp	14.29%	Amp	6.00%	
17	q25.3	Amp	28.57%	Amp	8.00%	
X	q12	Amp	17.14%	Amp	5.00%	
X	q28	Amp	40.00%	Amp	7.00%	

CNA, copy number alteration; ccRCC, clear cell renal cancer; TCGA, the Cancer Genome Atlas; Chr, chromosome; Del, delete; LOH, loss of heterozygosity; Amp, amplification.

Supplementary Table 2 Comparison between different T stage affected by copy number alteration.

Region	Event	T1	T2	T3	P-value	P-value
		(N=28)	(N=6)	(N=1)	T1 vs. T2	T1 vs. T2+T3
3p26.3-p11.1	Del	21	5	1	0.662	0.546
3p14.1	Del	15	5	1	0.179	0.121
3p12.2	Del	9	3	1	0.406	0.221
3q26.1	Del	15	1	1	0.1	0.237
5q31.2	Amp	15	3	0	0.874	0.612
5q35.1	Amp	15	3	1	0.874	0.865
7p11.2	Amp	11	3	0	0.628	0.863
7q31.2	Amp	8	1	1	0.549	1
8q11.21	LOH	16	4	1	0.667	0.49
11p11.2-p11.12	Amp	9	2	0	0.955	0.856
13q14.2	Amp	8	2	1	0.816	0.466
16p11.2-p11.1	LOH	28	6	1	-	-
17q21.33	Amp	9	2	0	0.955	0.856
19q13.2	Amp	9	0	1	0.105	0.35
20q11	Amp	7	0	0	0.169	0.139
20q11	Del	6	0	1	0.211	0.673
22q11.23	Del	14	2	1	0.458	0.735
Xp22.33	Amp	18	3	1	0.513	0.726
Xq28	Amp	13	1	0	0.179	0.121

Del, delete; LOH, loss of heterozygosity; Amp, amplification.

Supplementary Table 3 Comparison on affected genes burden among different T stage patients.

T stage	N	CN gain		CN loss		LOH		Total CNA	
		Mean	P-value	Mean	P-value	Mean	P-value	Mean	P-value
T1	28	2488		1176		2299		5963	
T2+T3	7	2603	0.43	2456	0.059	2013	0.732	7071	0.199

CN gain, copy number gain; CN loss, copy number loss; LOH, loss of heterozygosity; CAN, copy number alteration.

Supplementary Table 4 Comparison on affected bp per Mbp regions in whole genome (about 3000Mbp).

T stage	N	CN gain		CN loss		LOH		Total CNA	
		Mean	P-value	Mean	P-value	Mean	P-value	Mean	P-value
T1	28	91039		49700		71493		212232	
T2+T3	7	100449	0.586	55759	0.505	73198	0.531	229406	0.32

bp, base pair; Mbp, million bp; CN gain, copy number gain; CN loss, copy number loss; LOH, loss of heterozygosity; CAN, copy number alteration.
